# Optimization Model of Phenolics Encapsulation Conditions for Biofortification in Fatty Acids of Animal Food Products

**DOI:** 10.3390/foods10040881

**Published:** 2021-04-17

**Authors:** Roberta Tolve, Fernanda Galgano, Nicola Condelli, Nazarena Cela, Luigi Lucini, Marisa Carmela Caruso

**Affiliations:** 1 School of Agricultural, Forestry, Food and Environmental Sciences (SAFE), University of Basilicata, Viale Dell’Ateneo Lucano 10, 85100 Potenza, Italy; roberta.tolve@unibas.it (R.T.); nicola.condelli@unibas.it (N.C.); nazarena.cela@unibas.it (N.C.); marisa.caruso@unibas.it (M.C.C.); 2 DiSTAS—Department for Sustainable Food Process, Catholic University of the Sacred Heart, 29122 Piacenza, Italy; luigi.lucini@unicatt.it

**Keywords:** biofortification, central composite design, condensed tannins, dairy products, fatty acids profile, gum Arabic, maltodextrin, microencapsulation, milk, phenolic compounds

## Abstract

The nutritional quality of animal products is strongly related to their fatty acid content and composition. Nowadays, attention is paid to the possibility of producing healthier foods of animal origin by intervening in animal feed. In this field, the use of condensed tannins as dietary supplements in animal nutrition is becoming popular due to their wide range of biological effects related, among others, to their ability to modulate the rumen biohydrogenation and biofortify, through the improvement of the fatty acids profile, the derivate food products. Unfortunately, tannins are characterized by strong astringency and low bioavailability. These disadvantages could be overcome through the microencapsulation in protective matrices. With this in mind, the optimal conditions for microencapsulation of a polyphenolic extract rich in condensed tannins by spray drying using a blend of maltodextrin (MD) and gum Arabic (GA) as shell material were investigated. For this purpose, after the extract characterization, through spectrophotometer assays and ultra-high-performance liquid chromatography-quadrupole time-of-flight (UHPLC-QTOF) mass spectrometry, a central composite design (CCD) was employed to investigate the combined effects of core:shell and MD:GA ratio on the microencapsulation process. The results obtained were used to develop second-order polynomial regression models on different responses, namely encapsulation yield, encapsulation efficiency, loading capacity, and tannin content. The formulation characterized by a core:shell ratio of 1.5:5 and MD:GA ratio of 4:6 was selected as the optimized one with a loading capacity of 17.67%, encapsulation efficiency of 76.58%, encapsulation yield of 35.69%, and tannin concentration of 14.46 g/100 g. Moreover, in vitro release under varying pH of the optimized formulation was carried out with results that could improve the use of microencapsulated condensed tannins in animal nutrition for the biofortification of derivates.

## 1. Introduction

The growth of consumers’ concerns about animal origin product, combined with the demand for healthier foods, has increased researchers’ effort to develop safe and potentially health-promoting products. Besides the possibility to produce healthier animal origin product by adding specific nutrients in food through fortification, the increase of the concentration of active compounds supplementing animal feed with specific nutrients is getting attention [1]. In ruminants, the possibility to increase the unsaturated fatty acids, and particularly conjugated linoleic acid (CLA) via feeding strategies, is of particular interest.

Condensed tannins are a class of phenolic compounds with antioxidant, antibacterial, and rumen modulating properties that can inhibit the fatty acids biohydrogenation [2]. Some in vitro and in vivo studies have suggested that the supplementation of ruminant feed with these compounds may be an efficient tool to enhance the accumulation of vaccenic acid due to an inhibition of the last step of biohydrogenation on linoleic and linolenic acid [1,3]. Subsequently, the D—desaturase enzyme, acting on vaccenic acid, produces CLA, active in the prevention of cancer and atherosclerosis in mammals, so increasing its concentration in animal derivative food products [4]. Moreover, the supplementation of animal feed with condensed tannin potential increases the digestive utilization of dietary protein due to their ability to bind proteins under rumen pH conditions (preventing their excessive microbial degradation) and release them in the acid pH of the abomasum and in the alkaline conditions of the distal small intestine promoting, in this way, the protein digestion and absorption [3]. In addition, a significant increase in total phenolic compounds, and even an increase in antioxidant activity, has been reported in animal fed with polyphenolic compounds [5]. However, the bitter taste and the adverse effects of tannins on feed represent the major limitation to the practical application of this nutritional strategy to biofortify animal food derivates, as it leads to a reduction in voluntary dry matter intake by animals [6]. For this reason, it has been reported that the administration of encapsulated tannins instead of parent compound, could be a useful tool to overcome this drawback improving, at the same time, the bioavailability of the compound through a site-specific release in the rumen. Tannin microencapsulation would ensure a gradual release in the rumen that could improve tannin utilization in ruminant diets [6,7]. Specifically, the microencapsulation process consists of enclosing the active agent (namely core) in one or more polymeric matrix (namely shell) to protect it from light, oxygen, or other environmental factors, promoting its controlled release and masking its taste [8].

Spray-dryer technology, which converts a liquid solution containing the core and the shell material into a dry powder, is preferred due to its easy scalability and relatively low-cost process [9]. Different shell materials can be used for the encapsulation of plant extracts, although the limitation in term of suitability and cost must be considered, concerning the commercial use of microcapsules in livestock applications. Maltodextrin (MD) a hydrolyzed starch product that is odorless, colorless, and tasteless, is commonly used as an encapsulant in spray drying and has recently been used for the successful encapsulation of olive pomace polyphenols [10], chokeberry polyphenols [11], and citrus by-product extract [12]. In this field, MD has the advantage of being cheap, having low viscosity at high solid concentration and being able to protect the core material from oxidation. Similarly, gum Arabic (GA), a natural colorless plant heteropolysaccharide, has been commonly used as shell material in spray-drying, due to its interesting emulsifying and rheological properties, and besides its high protection against oxidation [13].

In the light of this, the objective of the present study was the microencapsulation of a polyphenolic extract rich in condensed tannins by spray-drying, using MD and GA as shell materials. A design of experiment based on a central composite design (CCD) model was performed. Furthermore, the release of phenolic compounds from the optimized formulation was evaluated at pH 5.6, 2.2, and 7.2, simulating the rumen, abomasum and intestinal conditions. The kinetic release was then studied with various computational models. Choosing the one that better fitted our results, it was possible to identify the phenomenon responsible of phenolic compounds released from microcapsules.

## 2. Materials and Methods

### 2.1. Materials

Quebracho (*Schinopsis* spp.) phenolic compound extract rich in condensed tannins (BYPRO Q) was kindly provided by Silvateam (S. Michele Mondovì, CN, Italy). Maltodextrin 16 DE and gum Arabic supplied by Tecnoblend (Matera, Italy) and Ingredion (Hamburg, Germany), respectively were employed as coating materials. Ultra-pure water (Merck- Millipore, Billerica, MA, USA) was used for analysis whereas distilled water was used for the preparation of microcapsules. All the other chemicals were of analytical grade and purchased from Sigma-Aldrich (Milan, Italy).

### 2.2. Characterization of the Tannin Rich Phenolic Extract by Spectrophotometric Assays

The Quebracho powder (QP), that is the phenolic compound extract rich in tannin, was characterized in terms of total phenolic compounds (TPC), total flavonoid content (TFC), and condensed tannin content (CTC). After weighing 0.2 g of QP in a 50 mL conical centrifuge tube covered with aluminum foil, 20 mL of a solution EtOH:H_2_O 60:40 (*v*/*v*) was added and the extraction was carried out using at 170 rpm an orbital shaker (Thermolyne AROS 160, Barnstead International, Boston, MA, USA) overnight at room temperature. After the extraction, the solution was centrifuged for 10 min at 3000× *g*, the recovered supernatant was filtered at 0.45 μm and analyzed to quantify through spectrophotometric assays the TPC, TFC, and CTC. TPC in QP extract were determined as described by Singleton and Rossi [14]. Briefly, 100 μL of the extract were incubated with 500 μL of ten times diluted Folin–Ciocalteau reagent for 10 min at room temperature. Successfully, 500 μL of a saturated solution of Na_2_CO_3_ were added. After 30 min in the dark at room temperature, the absorbance was measured at 765 nm (Cary 1E UV–VIS spectrophotometer, Varian, Agilent, Milano, Italy). Gallic acid was employed as calibration standard and results were expressed as mg gallic acid equivalents (GAE)/g dw. The total flavonoid content (TFC) of the extract was evaluated according to Dewanto et al. [15]. To 100 μL of the extract, 440 μL of 0.066 M NaNO_2_ solution and, after 5 min at room temperature, 60 μL of AlCl_3_ 0.75 M were added. Finally, after 6 min, 400 μL of NaOH 0.5M were added and the absorbance at 500 nm was measured. Catechin was employed as calibration standard and results were expressed as mg of catechin equivalents (CAE)/g dw. The condensed tannin content (CTC) of the extract was determined by the vanillin assay as reported by Caruso et al. [16]. In detail, 200 μL of the extract was mixed with 800 μL of vanillin reagent (containing 3% of vanillin and 14% of HCl in MeOH) and allowed to react for 20 min at room temperature. The absorbance was recorded at 500 nm and the results were expressed as mg catechin equivalents (CAE)/g dw.

### 2.3. Characterization of the Tannin Rich Phenolic Extracts by UHPLC-ESI/QTOF Mass Spectrometry

Aqueous and EtOH:H_2_O 60:40 (*v*/*v*) extracts were produced dissolving 0.5 g of QP in the specific solvent. After the centrifugation for 10 min at 3000× *g*, aqueous and EtOH:H_2_O extracts were filtered through a 0.22 μm cellulose syringe filter into HPLC amber glass vials. Phenolic compounds were then profiled according to an untargeted approach using liquid chromatography coupled to quadrupole-time-of-flight mass spectrometry (UHPLC-ESI/QTOF-MS) as previously reported [17]. In brief, chromatographic separation was carried out using a binary linear gradient of aqueous acetonitrile (5 to 95% in 34 min) and a Zorbax Eclipse Plus column (50 mm × 2.1 mm, 1.8 μm—Agilent technologies, Santa Clara, CA, USA). The injection volume was 4 μL and QTOF-MS acquired high-resolution spectra in positive polarity and full scan mode (100–1200 *m*/*z*, 1 Hz,). Four replicate extracts were analyzed per extraction condition.

The annotation of mass features from raw data was done using the software Profinder revision B.07 (Agilent Technologies), by combining monoisotopic accurate mass isotopic spacing and isotopic ratio. Mass accuracy was <5 ppm and both mass and retention time alignment (0.1 min) were adopted [18]. The database Phenol-Explorer 3.6 (http://phenol-explorer.eu/: accessed on: 2 March 2021) was used to annotate phenolic compounds, and the compounds were retained only when annotated within 100% of replications within at least one extraction condition.

Values are calculated using calibration curves built from sesamin (lignans), ferulic acid (hydroxycinnamic acids and other phenolic acids), cyanidin (anthocyanins), catechin (flavanols), luteolin (flavones and other remaining flavonoids), resveratrol (stilbenes), and tyrosol (tyrosols and other remaining low molecular weight phenolics).

Phenolics were finally grouped in sub-classes (according to phenol-explorer classification), and the cumulative abundances from each class used for quantification purposes. To this aim, calibration curves prepared from solutions made from pure standard solutions (Extrasynthese, Lyon, France), were used [18].

### 2.4. Microparticles Production

In order to obtain the microencapsulated tannins, maltodextrin (MD) and gum Arabic (GA), the selected coating materials, were separately weighed and rehydrated in distilled water under magnetic stirring overnight at room temperature and finally homogenized. After mixing the MD and GA solution, with a total solid concentration of 10% (*w*/*v*), QP was added under continuous stirring and homogenized for 3 min at 9500 rpm with an Ultra Turrax T25 homogenizer (IKA Instruments, Staufen, Germany). Detailed information about the MD:GA and core:shell ratio is shown in Table 1 according to CCD. Afterwards, the obtained solutions were atomized using a spray dryer (FT 80 Tall Form spray dryer, Armfiled Inc., Jackson, NJ, USA). The spry-dryer operative conditions, selected based on the available literature and our previous experiments, were as follows: inlet temperature of 170 °C, outlet temperature ranged from 60 to 65.5 °C, pump setting of 0.5 L/h, air flow of 600 L/h, and nozzle cup diameter of 0.7 mm.

### 2.5. Microparticles Characterization

#### 2.5.1. Encapsulation Yield

The encapsulation yield (EY) was calculated as the ratio between the powder collected at the bottom of the spray-dryer’s cyclone separator and the amount of the initial solids contained in the feed suspensions Equation (1).

(1) $$\mathrm{EY}=\frac{\mathrm{Mass}\;\mathrm{of}\;\mathrm{powder}\;\mathrm{collected}\;\left(g\right)}{\mathrm{Mass}\;\mathrm{of}\;\mathrm{solid}\;\mathrm{fed}\;\left(g\right)}\times 100$$

#### 2.5.2. Encapsulation Efficiency and Loading Capacity

Two different extractions protocols were employed to quantify total phenolic content and the surface phenolic content of microcapsules required for the evaluation of the encapsulation efficiency (EE) and the loading capacity (LC) as reported by Zanoni et al. [19]. Detailed, to obtain the total phenolic content, the rupture of the capsules and their release was granted suspending in 1 mL of water 100 mg of the powder and placing the tube in a sonication bath at room temperature for 30 min. Successfully, 10 mL of ethanol were added and the mixture was left under magnetic stirrer for 30 min. As regards the surface phenolic content, 100 mg of microcapsules were incubated in 10 mL of ethanol for 1 min. The obtained extracts were centrifuged at 4500× *g* for 10 min and finally, the TPC and CTC were assessed. The LC and EE were calculated as reported by Xu et al. [20]:

(2) $$\mathrm{LC}=\frac{\mathrm{Total}\;\mathrm{phenolic}\;\mathrm{content}-\mathrm{Surface}\;\mathrm{phenolic}\;\mathrm{content}}{\mathrm{Mass}\;\mathrm{of}\;\mathrm{microparticles}}\times 100$$

(3) $$\mathrm{EE}=\frac{\mathrm{Total}\;\mathrm{phenolic}\;\mathrm{content}-\mathrm{Surface}\;\mathrm{phenolic}\;\mathrm{content}}{\mathrm{Theoretical}\;\mathrm{phenolic}\;\mathrm{content}\;}\times 100$$

In addition, TC (g/100 g) namely tannin content was evaluated.

#### 2.5.3. Moisture Content and Water Activity

The moisture content was evaluated based on AOAC method [21]. Instead, the water activity (a_w_) of the spray dried microcapsules was measured by using a HygroPalm with an HC2-AW sensor (Rotronic Italia Srl, Milano, Italy) at 25 °C.

#### 2.5.4. Color Analysis

The color was measured by a reflectance colorimeter (illuminant D65) (Minolta Chroma meter CR-300, Osaka, Japan) based on the color system CIE—L *, a *, b *. Specifically, lightness (L *) and color (+a: red; −a: green; +b: yellow; −b: blue) of QP and of microcapsules were assessed.

#### 2.5.5. Optical Microscopy

After the suspension in mineral oil, the shape and the size of the microparticles was observed by a digital light microscope (Nikon Eclipse 80i) at 100× magnification and with the ImageJ software (version 1.53a) was used to set the scale-bar.

### 2.6. Design of Experiment (DOE) Using Central Composite Design (CCD) and Response Surface Methodology (RSM)

A central composite design (CCD) approach was applied to optimize the formulation parameters of QP microencapsulation, requiring a minimum of experiments. Specifically, a 2 factor 3 level CCD approach-based on response surface methodology (RSM) analysis was used. The core:shell ratio (X_1_) and the MD:GA ratio (X_2_) were selected as independent variables and studied at three different levels coded as −1, 0, and 1. Moreover, five central repetitions were realized, resulting 13 experiments with the α value of orthogonality equal to ±1.14, as presented in Table 1. Encapsulation yield (Y_1_), encapsulation efficiency (Y_2_), loading capacity (Y_3_), and tannin content (Y_4_) were selected as dependent response variables to be optimized. The regression coefficients (β) were obtained by adapting the experimental results to a second-order polynomial model Equation (4):

Y_k_ = β_0_ + β_1_ X_1_ + β_2_ X_2_ + β_12_ X_1_ X_2_ + β_11_ X_1_^2^ + β_22_ X_2_^2^ (4)

where, Y_k_ is the response variable, X_1_ and X_2_ are the levels of the independent variables core:shell and MD:GA, respectively. β_0_ is a constant, β_1_, β_2_ are the linear coefficients, β_11_ and β_12_ are the quadratic coefficients of the model.

### 2.7. In Vitro Kinetics Release of Encapsulated Tannin from Microcapsules

The in vitro kinetic release in the digestive system of ruminant animals of not encapsulated-QP and QP encapsulated under the optimal conditions was simulated following the procedure of Adejoro et al. [6]. To simulate the rumen, abomasal, and intestinal conditions the acetate buffer (pH 5.6), HCl buffer (pH 2.2), and phosphate buffer (pH 7.4) were used, respectively. Two hundred microgram of the encapsulated and the not encapsulated QP were suspended in 50 mL of elution media and agitated at 50 rpm. An aliquot (1 mL) of sample was taken at 0.5, 1, 2, 4, 8, and 24 h, and replaced with an equivalent volume of the corresponded elution media. The release of TPC was monitored by UV spectrophotometry as described above. The cumulative amount of TPC at each time interval was corrected with the volume of the elution media. To find the best model for the TPC released in rumen, abomasum, and intestinal environment, the release kinetics of TPC were calculated using different models reported by Navarro-Flores et al. [22]:

zero order release Qt = Q_0_ + k_0_t (5)

first order release logQt = logQ_0_ − k_1_t (6)

Higuchi model Qt = Q_0_ + k_H_t^1/2^ (7)

where k_0_ is the zero-order rate constant, t is the time, Qt is the released concentration of phenolic compounds at time t, Q_0_ is the initial concentration of phenolic compound within solutions (usually Q_0_ = 0), k_1_ is the first-order rate constant, and k_H_ is the Higuchi dissolution constant. Furthermore, to better characterize the mechanism of TPC release from microparticles, data were analyzed with the equation proposed by Korsmeyer and Peppas:

Qt = k_KP_tn (8)

where k_KP_ is the proportionality constant and n is the release exponent that could be used to indicate the mechanism of release [23].

### 2.8. Statistical Analysis

All data reported (i.e., mean values ± standard deviation) represent the means of at least three measurements. To optimize the microencapsulation process, dependent variables were analyzed using the central composite design and the lack of fit, coefficient of determination (R^2^), adjusted coefficient of determination (R^2^_adj_), model *p*-value, and the construction of response surface (3D) graphs were obtained using the software XLSTAT Premium (Version 2019.4.2, Addinsoft SARL, Paris, France). The optimized formulation was obtained using the software’s desirably function. The independent variables were kept within range while the responses were maximized according to the process requirement. The general approach of the desirability function is to transform the response (Y) into a dimensionless individual desirability function. The scale of the desirability function ranges between 0 (completely undesirable response) and 1 (fully desirable response). The overall desirability for maximum response value and variables was fixed to obtain the desired criteria.

## 3. Results and Discussion

### 3.1. Characterization of the Quebracho Tannin Rich Phenolic Extracts

Quebracho powder (QP) was characterized through spectrophotometer assays in terms of TPC, TFC, and CTC before being microencapsulated. The powder was made up for more than 84% of TPC (84.37 ± 0.72 g GAE/100 g of powder) with CTC that represent the largest part of these (68 ± 2.16 g CAE/100 g of powder) and TFC the smallest (15.76 ± 1.60 g CAE/100 g of powder), results broadly in line with Marsal et al. [24] who characterized the same tannin extract.

### 3.2. UHPLC-ESI/QTOF Phenolic Profiling

The metabolomics approach allowed highlighting a broad diversity of phenolics in our extracts, that included more than 400 compounds ascribable to flavonoids, hydroxycinnamic acids, and other phenolic acids, tyrosols, and other low molecular weight phenolics, as well as lignans and stilbenes. The whole list of phenolic compounds annotated in either aqueous or ethanolic extracts is provided as Supplementary Materials, according to the phenolic sub-class and together with individual abundance values and composite mass spectra (monoisotopic mass and abundance combinations).

**Figure 1:**
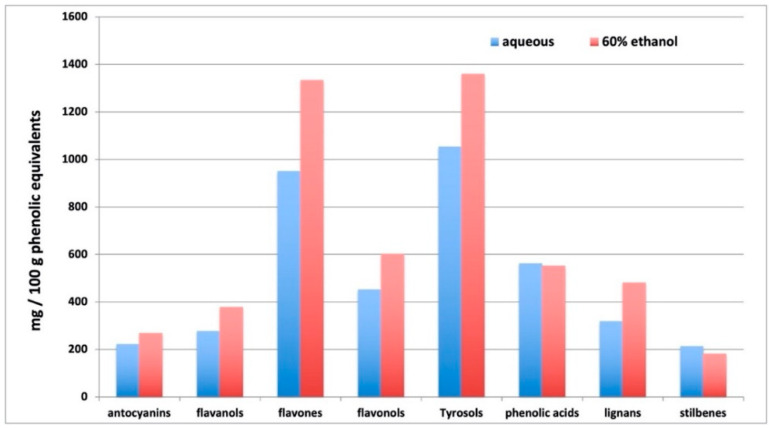
Cumulative semi-quantitative abundance (mg/100 g phenolic equivalents) of the different phenolic subclasses, as profiled by untargeted metabolomics in aqueous and EtOH:H_2_O 60:40 extracts.

As expected, the ethanolic solution was generally more effective in extracting phenolics, even though phenolic acids did not change (fold-change 0.98) and stilbenes decreased in ethanolic solution (fold-change = 0.85). In more detail, flavones were the most represented, ranging from 950 to 1334 mg/100 g in aqueous and ethanolic extract, respectively, together with tyrosol equivalents (alkylphenols, tyrosols, phenolic terpenes, and other low molecular weight phenolics—1053 to 1360 mg/100 g in aqueous and ethanolic extract, respectively). Among flavone equivalents, flavanones (naringenin and naringin derivatives), apigenin and luteolin conjugates, and isoflavonoids (daidzein and genistein derivatives, among others) were the most represented. Notwithstanding, the extracts were rich in anthocyanins such as cyanidin, malvidin and petunidin glycosides, flavanols, proanthocyanidins dimers and trimers, lignans and stilbenes were present in appreciable amounts. The whole phenolic profile is summarized in Figure 1, where cumulate abundances per each phenolic sub class are provided as a function of the extraction solvent.

### 3.3. Experimental Data for Process Optimization

According to the possible microcapsule’s application, the effect of the core:shell and the MD:GA ratio on the encapsulation yield, total and surface phenolic compounds, encapsulation efficiency, loading capacity, tannin content, a_w_, moisture, and color of microencapsulated QP was evaluated and the results are reported in Table 2.

#### 3.3.1. Encapsulation Yield

Encapsulation yield (EY) ranged from 29.75% to 48.92%. Given the sparse literature dealing with microencapsulation of this extract, these data cannot be readily compared to the semi-technical pilot plant spray dryer used here.

In particular, the larger size of the spray-dryer may have resulted in greater adherence of the powder particles to the walls of the drying chamber, and thus a lower EY. The highest EY (experiment No. 3, 4, and 8) was obtained with the increase of the amount of MD as shell material at the expense of GA concentration. At the same time, it was found that the lowest EY was observed in the samples with higher GA concentration (experiment No. 1, 2, and 7) up to a value of 29.75% in the sample with MD:GA ratio of 1.5:3.5. Therefore, it can be concluded that the increasing ratios of MD:GA have a negative effect on the yield. The same conclusion was reached by Tolun et al. [25], who investigated the influence of different MD:GA ratio (10:0, 8:2, and 6:4) on the microencapsulation of grape polyphenols using spray dryer. This could be due to the short-chain branched structure of GA and to its high hydrophilicity which probably increased the adhesion of the particles to the chamber of the spray and to other dried particles [26].

#### 3.3.2. Encapsulation Efficiency and Loading Capacity

Total phenolic compound and the surface phenolic compounds are parameters used to determine the encapsulation efficiency (EE) and the loading capacity (LC). These values reflect the percentage of the phenolic compounds successfully entrapped in the microcapsules (EE) and the amount of loaded phenolic compounds per unit weight (LC). In contrast to the total phenolic compounds, which yielded a higher correlation with the core:shell ratio (R^2^ = 0.99), the correlation with the surface phenolic compounds was low (R^2^ = 0.69), indicating that the surface phenolic compounds did not increase linearly with the increase of QP in the formulation. This was probably due to the ability of the different MD:GA ratio to entrap the phenolic compounds, which varied the amount of phenolic compounds on the surface of the microcapsules. The values of TPC and surface phenolic compound values allowed the calculation of the EE. According to Mahdavi et al. [13] a successful encapsulation method dealing with the high retention of the core materials and minimum amounts of the core on the surface. The EE, which ranged from 68.03 to 76.74%, was strongly influenced by both core:shell and MD:GA ratio. In particular, the results showed that the 2:3 MD:GA and 1.5:5 core:shell ratio (experiment No. 2) increased the EE to a value of 76.58% which was close to the highest value obtained with the 1.5:3.5 MD:GA and 1:5 core:shell ratio (experiment No. 7). Therefore, it can be concluded that with the increase in the proportion of GA in the shell material an increase in the EE occurred. This effect could be caused by the emulsifying properties of GA and its ability to form a dried matrix that prevents the core material from contacting the environment, as reported by Cilek et al. [27] who observed a significant increase in EE of phenolic compounds extracted from cherry pomace with increasing MD:GA ratio.

Furthermore, the higher EE obtained increasing the GA concentration could be related to the different viscosity of the feed suspension.

As reported by Premi et al. [28], the feed suspension viscosity increased as a function of GA concentration. This implies a lower volume of water to be evaporated and thus a shorter time needed to form a crust, which reduces the circulation movements within the droplets and leads to a higher retention of the active material [29]. However, the combination of two different shell materials (MD:GA) is confirmed to be a suitable choice for the microencapsulation of phenolic compounds. About it, Tolun et al. [25] have reported that the use of a mixture of MD:GA as shell material for the encapsulation of phenolic compounds extracted from grape residue was more appropriate than the use of MD alone in terms of EE.

The LC value ranged from 3.84% to 17.76% and tended to improve with the increase of MD:GA and core:shell ratio. In particular, the higher values were reported in the experiment No. 2 and 6. A similar trend was observed for tannin content, although it must be emphasized that the formulations obtained in experiment No. 2 and 6 differed for both core:shell (1.5:5 vs. 1.7:5) and MD:GA (2:3 vs. 2.5:2.5) ratio. With respect to the core:shell ratio, these results suggest that it may be not useful to further increase the amount of QP in the formulation exceeding the 1.5:5 ratio. Moreover, as confirmed by the surface phenolic compounds, no higher amount of QP was encapsulated in experiment No. 6 compared to No. 2.

#### 3.3.3. Moisture Content and Water Activity

Moisture content and a_w_ are important indices of powders from spray drying process as they affect its stability, agglomeration, and shelf-life. The moisture content of QP microcapsules was less than 5% in all formulations, meeting the requirements for the moisture content of a food powder (<6%) and suitable for long-term storage [30,31]. In detail, the moisture content agreed with microcapsules obtained under similar operating conditions [9] and ranged from 3.32 to 4.34%, tending to decrease with the increase of GA concentration. These results are not entirely consistent with those obtained by other authors [32,33]. For example, Kang et al. [32] reported that the moisture content of chlorophylls microcapsules decreased with an increase in MD concentration. However, it must be underlined that the differences between formulations could be related to the MD and GA chemical structure, especially the hydrophilic groups and ramification that can bind water molecules, but also depend on the MD degree of polymerization [26]. Rodríguez-Hernández, et al. [34], verified higher moisture contents for the powders produced by spray-drying with MD 10DE than for those produced with MD 20 DE. The authors attributed this difference to the polymerization degree of each agent and concluded that moisture retention was greater for MD 10 DE due to its better binding properties. Thus, our results could be due to the MD degree of polymerization used here (16DE) which is not specified elsewhere [32,33]. The a_w_ content ranged from 0.34 to 0.44, such values suggest that QP microcapsules could be considered stable materials concerning microbial contamination [31,35].

#### 3.3.4. Color Analysis

The color parameters of QP were characterized by values of 32.18 for L *, 27.15 for a *, 13.67 for b *. When these values were compared with the microencapsulated QP, a greater increase in L * and b * and a decrease in a * associated with lightness, yellowness, and redness, respectively, were found.

This could be explained by the white color of MD and the white/yellowish color of GA. These results were expected and are in agreement with the literature [27]. The different formulations obtained were characterized by a significant difference with respect to the parameters L *, a *, and b *. In particular, the color change was correlated with the core:shell ratio with an R^2^ of −0.81, 0.82, and 0.77 for L *, a *, and b *, respectively. Thus, increasing the core:shell ratio a decrease in lightness, and an increase in redness and yellowness was observed.

### 3.4. Model Fitting and Statistical Verification

To the best of our knowledge, a blend of MD and GA has already been examined for the microencapsulation of a phenolic extract rich in tannin using freeze-dryer technology by Adejoro et al. [6]. The approach used by the researchers was the so-called “one variable at time” method in which it was possible to consider just one variable while the others remain constant. The disadvantages of this method are ignoring factors interactions, the relatively high number of experiments, and time/reagent consuming approach [13].

To optimize the formulation of the microparticles, central composite design (CCD) was achieved considering linear, quadratic, and interaction effects between core:shell (X_1_) and MD:GA ratio (X_2_) selected as independent variables, on QP microencapsulation. By varying the selected independent variables and replicating five times the central point, 13 spray-dried powders were obtained and Table 2 shows the results of the responses. A second-order polynomial model, described by Equation (4), was fitted to the experimental data values obtained for each response variable studied. The determination coefficients (R^2^ and R_Adj_^2^), and the linear and quadratic effects of the factors, as well as their interaction, the lack of fit, and the significance of the model for each response variable are presented in Table 3.

The results showed that the mathematical model used allows to obtain good determination coefficients; in fact, according to Corrêa-Filho et al. [36], values above 0.7 indicate that the fitted equations adequately describe the effects of core:shell and MD:GA ratio on each dependent variable. In detail, the calculated models explained 99.5%, 79%, 77.8%, and 99.7% of the results for LC, EE, EY, and TC, respectively. Additional confirmation of model validity is given by the lack-of-fit *p*-values for the different equation model, which are all insignificant (*p* > 0.05), indicating that the models can adequately fit the experimental data. LC, EE, EY, and TC were significantly influenced by the linear and quadratic terms of the core:shell ratio (*p* < 0.05). All the independent variables, except EY, were significantly influenced by the quadratic terms of MD:GA ratio and none of the dependent variables evaluated was dependent by the interaction between the two variables (*p* > 0.05). Moreover, a significant effect of the linear and quadratic terms of both the core:shell and the MD:GA ratio whereas a negligible effect of their interaction was observed for the TC. In detail, a negative quadratic effect of X_1_ on the independent variables was observed, indicating that the response variables peak was achieved at a certain core:shell and MD:GA ratios and diminishing with further increases in the core:shell and MD:GA ratios. This suggests that high core:shell and MD:GA ratio is not beneficial to further improve the microencapsulation process.

The final equations of LC, EE, EY, and TC were coded as follows:

LC = 12.292 + 5.193 X_1_ − 0.188 X_2_ − 0.688 × X_1_^2^ + 0.197 X_2_^2^ (9)

EE = 73.484 + 0.773 X_1_ − 1.098 X_2_ − 1.947 × X_1_^2^ + 0.986 X_2_^2^ (10)

EY = 38.749 + 1.129 X_1_ + 5.366 X_2_ − 1.181 X_1_^2^ (11)

TC = 98.665 + 43.613 X_1_ − 1.863 X_2_ − 2.552 X_1_^2^ + 1.101 X_2_^2^ (12)

**Figure 2:**
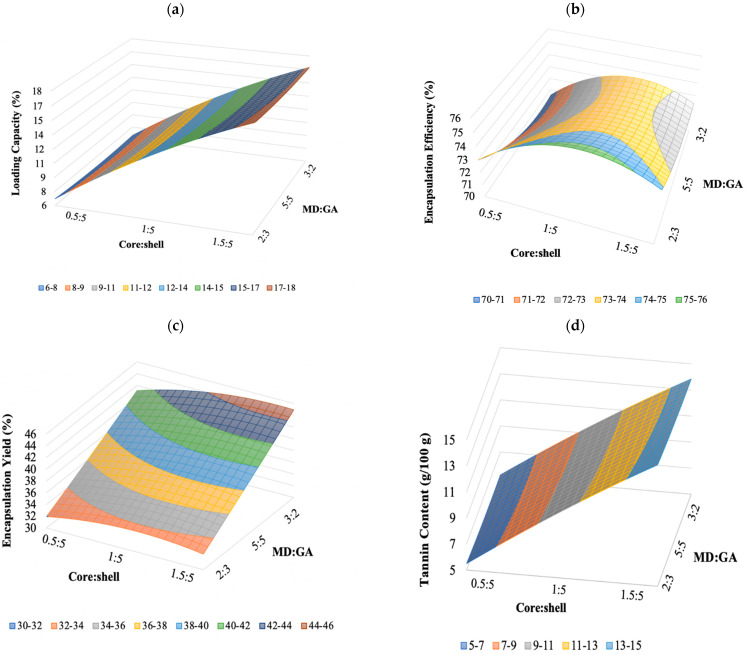
Response surfaces (**a**–**d**) showing influence of core:shell and MD:GA on LC (**a**), EE (**b**), EY (**c**), and TC (**d**).

To visualize the relationship between the response and experimental levels of the independent variables, 3D surface response plots were constructed according to the quadratic polynomial model Equations (9)–(12) (Figure 2a–d). The surface plots are a very useful tool in examining the main effect and interaction effects of two or more factors. The plots were created by plotting the response against two independent variables using the *z*-axis. The 3D plot of the response surface of regression Equation (9) showed the effects of the core:shell and MD:GA ratio and their interaction on the LC (Figure 2a). Accordingly, the LC increased with the increase of core:shell ratio at any given MD:GA ratio similarly to TC as shown by the plot of the regression Equation (10) in Figure 2b. Instead, the response plot in Figure 2b,c, related to the Equations (11) and (12), respectively showed that was improved with an increasing amount of MD:GA and a core:shell ratio close to 1:5 EE instead the EY was higher with a core:shell ratio of 1.5:5 and MD:GA of 3:2.

The optimal zone in which every point represented a combination of the core:shell and MD:GA ratio gave the maximum values of all the responses.

**Figure 3:**
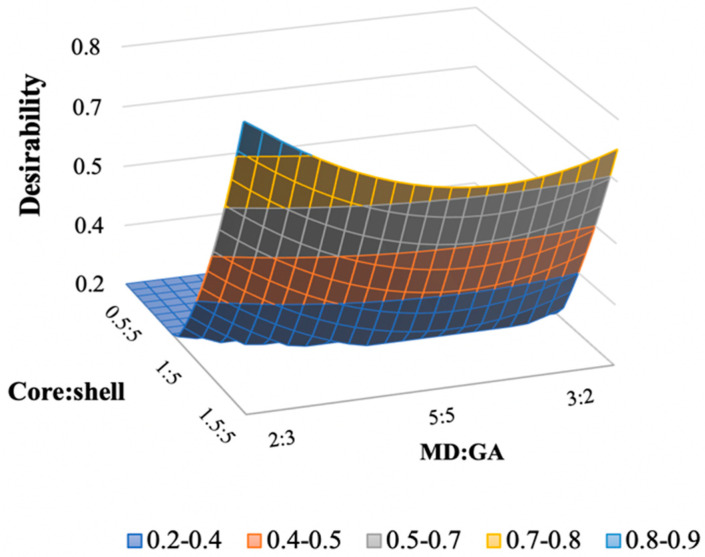
Response surfaces plot of desirability for the studied core:shell and MD:GA ratio.

According to Derringer and Suich [37], the desirability approach was determined using the responses LC, EE, EY, and TC, as well as the model parameters determined using the CCD. The highest desirability was determined by assigning the maximum level of the variables and choosing as factor settings the studied parameters. In this study, both the core:shell and MD:GA ratio have a significant effect on the QP microencapsulation process. Thus, optimization allows to obtain a formulation with the desired characteristics concerning all the responses. Microencapsulation of QP was optimized considering the maximization of the microencapsulation parameters namely loading capacity, encapsulation efficiency, encapsulation yield, and tannin content. Maximum desirability that can be achieved is 1. Desirability above 0.8 is represented by light blue colored region of the contour plot. The desirability increased with the increase of the core:shell ratio and with a MD:GA ratio close to 2:3 as evident from Figure 3. Desirability surface plot combines the individual surface plots of the responses based on the desirability criteria set for each.

The optimized formulation developed in this study had a core:shell ratio of 1.5:5 and a MD:GA ratio of 2:3, corresponding to the highest desirability of 0.9 as shown in Figure 3.

### 3.5. Optical Microscopy

**Figure 4:**
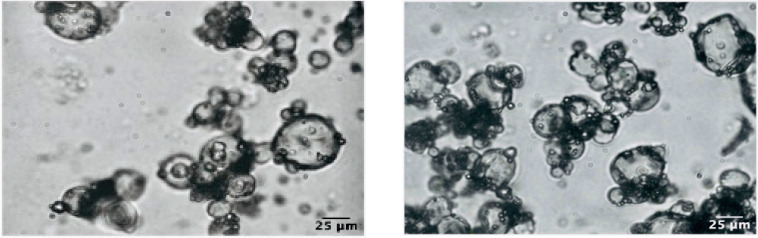
Microphotographs of the optimized microencapsulate QP at 100× magnification.

A typical light micrograph showing the shape and the size of the microparticles of the optimized microencapsulated QP powder was reported in Figure 4. As it is possible to observe, the particles size was much lower than 100 μm.

### 3.6. In Vitro Kinetics Release of Encapsulated Tannin from Microcapsules

**Figure 5:**
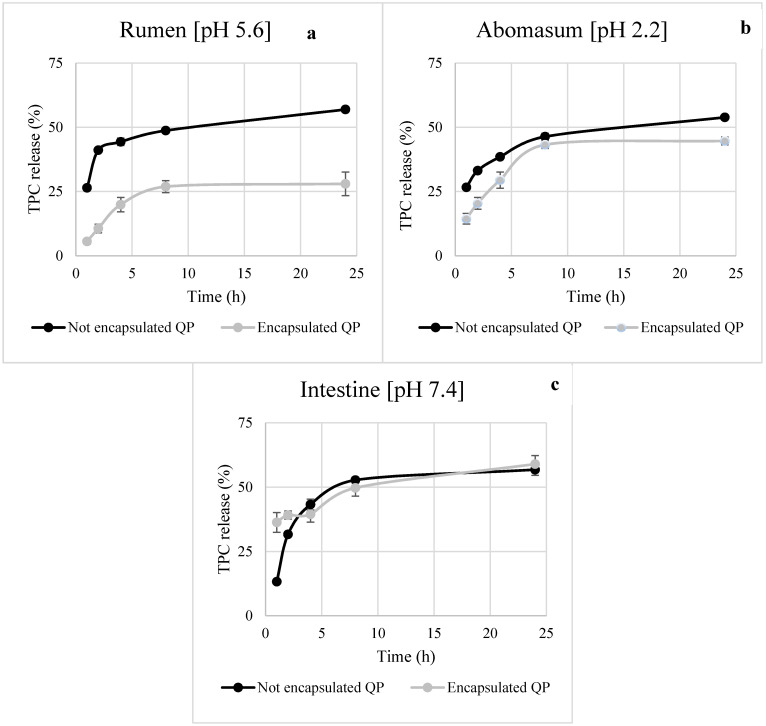
In vitro release profile of not encapsulated and encapsulated tannin extract in simulated rumen (**a**) abomasum (**b**) and intestinal (**c**) environment.

Controlled release of bioactive compounds at the right place and time is one of the more interesting functionalities that can be provided by microencapsulation. Timely and targeted release improves the efficacy of a bioactive compound and often its bioavailability [38]. The release profile of TPC from the not encapsulated QP and the encapsulated under optimal core:shell and MG:GA ratio in acetate, phosphate, and HCl buffer elution media was reported in Figure 5a–c. At pH 5.6 and 2.2, which simulated rumen and abomasum conditions, respectively, a strong reduction in TPC release from microencapsulated QP compared to the not encapsulated QP was observed. A different trend was recorded in the intestinal environment at pH 7.4, where a burst release pattern of TPC similar to not encapsulated extract was obtained. This result might be due to GA properties. In detail, it has been reported that GA tends to swell and disrupt the structure of GA microparticles in elution media with pH values higher than 6.5, resulting in more porous microparticles [39]. The intrinsic properties of the shell materials, the chemical interactions between the shell materials and the encapsulated compound, and the environment affect the release behavior. Since the modulation of the biohydrogenation by the condensed tannins contained in QP occurs in rumen, the microencapsulated form could allow a slow release and increase the bioavailability of compounds [6,7].

As widely reported, the quality of microparticles is closely related to their ability to retain the core material until the target site [40,41]. This ability can be evaluated by performing release tests in different elution media. Bearing this in mind, TPC release from not encapsulated and encapsulated QP was kinetically studied using the zero-order, first-order, Higuchi and Kors-Peppas models in the rumen, abomasum, and under simulated intestinal conditions (Table 4). The zero-order kinetic model, which is usually applicable to poorly soluble compounds, describes the phenomenon of slow-release, in a shell that does not disintegrate. In first-order kinetics, the dissolution of the compound, which is generally soluble in water and entrapped in porous shell material, is proportional to its concentration. The Higuchi model refers to the release kinetics involving both diffusion and dissolution, instead the Korsmeyer-Peppas model helps to understand the release mechanism of a drug and categorize it into Fickian diffusion, non-Fickian, Case II transport, and Super Case II transport [41]. Based on the correlation coefficients, for “not encapsulated QP” and “encapsulated QP,” in the rumen, abomasum, and intestinal environment the model that better fitted our data was Higuchi with a correlation coefficient between 0.7464 and 0.849, which specifically means that the released phenolic compounds flow unidirectionally from the encapsulating matrix to the release medium (Fick’s diffusion law), as reported by Quintal Martínez et al. [42]. A similar trend was reported by Adejoro et al. [6] who, when evaluating the tannin release from MD:GA microcapsules in rumen elution media, concluded that the Higuchi model best fitted the obtained results with an R^2^ ranging from 0.836 to 0.924. So, as reported by Norkaew et al. [38], the release rate of an encapsulated compound is influenced by the solubility of the different wall materials in the release environment which may, consequently, contribute to a faster or slower destruction of the microcapsules structure.

## 4. Conclusions

This study showed that the CCD and RSM could be useful as tools of optimization to realize microcapsules containing a phenolic extract rich in condensed tannin.

The four regression models were significant, and the lack-of-fits were insignificant. RSM predicted that the formulation obtained with a core:shell ratio of 1.5:5 and a MD:GA ratio of 2:3 provides the highest value for the microencapsulation process evaluated in terms of loading capacity, encapsulation efficiency, encapsulation yield, and tannin content. QP microencapsulated under the optimized conditions exhibited a slow release in acetate buffer, simulating the rumen environment. It is the indication that this formulation could be used as a feed supplement in ruminants nutrition aimed at biofortifying animal food products, hence increasing the concentration in unsaturated fatty acids.

## Figures and Tables

**Table 1:** Real and codified values used in the CCD.

Experiment No.	Actual Values		Coded Values	
	X_1_ Core:Shell	X_2_ MD:GA (*w*/*w*)	X_1_	X_2_
1	0.5:5	2:3	−1	−1
2	1.5:5	2:3	+1	−1
3	0.5:5	3:2	−1	+1
4	1.5:5	3:2	+1	+1
5	0.29:5	2.5:2.5	−1.414	0
6	1.7:5	2.5:2.5	+1.414	0
7	1:5	1.5:3.5	0	−1.414
8	1:5	3.5:1.5	0	+1.414
9	1:5	2.5:2.5	0	0
10	1:5	2.5:2.5	0	0
11	1:5	2.5:2.5	0	0
12	1:5	2.5:2.5	0	0
13	1:5	2.5:2.5	0	0

**Table 2:** Experimental results of quebracho extract microencapsulation by spray drying carried out according to the central composite design.

Experiment No.	Encapsulation Yield (%)	Total Phenolic Compounds (g/100 g)	Surface Phenolic Compounds (g/100 g)	Tannin Content (g/100 g)	Encapsulation Efficiency (%)	Loading Capacity (%)	Moisture	a_w_	Color		
									L *	a *	b *
1	32.48 ± 1.36	6.93 ± 0.16	0.32 ± 0.02	5.50 ± 0.09	72.64 ± 1.88	6.60 ± 0.17	3.35 ± 0.23	0.44 ± 0.00	76.78 ± 0.96	2.72 ± 0.23	22.89 ± 0.31
2	35.69 ± 0.53	18.24 ± 0.03	0.56 ± 0.00	14.46 ± 0.25	76.58 ± 0.16	17.67 ± 0.04	3.67 ±0.03	0.37 ± 0.00	71.08 ± 1.40	4.11 ± 0.27	27.13 ± 0.33
3	42.62 ± 0.49	6.62 ± 0.09	0.32 ± 0.04	5.26 ± 0.07	69.33 ± 1.10	6.30 ± 0.10	4.22 ± 0.02	0.41 ± 0.00	75.01 ± 1.26	3.02 ± 0.15	24.38 ± 0.14
4	48.92 ± 0.94	17.67 ± 0.06	0.58 ± 0.03	14.02 ± 0.37	74.09 ± 0.17	17.10 ± 0.04	4.30 ± 0.09	0.40 ± 0.00	70.35 ± 0.70	4.22 ± 0.08	27.20 ± 0.22
5	34.29 ± 0.85	4.10 ± 0.11	0.26 ± 0.00	3.200 ± 0.1.2	70.05 ± 1.93	3.84 ± 0.11	4.11 ± 0.06	0.36 ± 0.00	78.98 ± 0.46	2.27 ± 0.04	21.48 ± 0.13
6	33.96 ± 0.81	19.33 ± 0.04	1.58 ± 0.02	15.34 ± 0.29	68.03 ± 0.22	17.76 ± 0.06	4.19 ± 0.12	0.38 ± 0.00	69.18 ± 0.17	4.71 ± 0.03	28.38 ± 0.05
7	39.75 ± 0.87	13.14 ± 0.03	0.35 ± 0.02	10.29 ± 0.06	76.74 ± 0.03	12.79 ± 0.00	4.070 ± 0.24	0.34 ± 0.00	71.03 ± 0.08	4.24 ± 0.06 ^b^	27.37 ± 0.04
8	43.57 ± 1.18	12.72 ± 0.13	0.38 ± 0.01	9.71 ± 0.1.5	74.06 ± 0.82	12.34 ± 0.14	3.96 ± 0.14	0.37 ± 0.00	70.97 ± 1.09	4.13 ± 0.20	27.55 ± 0.41
9	38.42 ± 0.45	12.55 ±0.13	0.36 ± 0.05	9.95 ± 0.27	73.17 ± 0.46	12.20 ± 0.08	4.34 ± 0.07	0.38 ± 0.00	70.56 ± 0.49	4.28 ± 0.05	27.65 ± 0.11
10	37.80 ± 0.63	12.63 ± 0.04	0.35 ± 0.01	9.85 ± 0.16	73.67 ± 0.22	12.28 ± 0.05	3.97 ± 0.13	0.35 ± 0.00	70.01 ± 0.61	4.45 ± 0.16	28.02 ± 0.37
11	38.81 ± 0.82	12.69 ±0.07	0.37 ± 0.03	9.86 ± 0.14	73.92 ± 0.35	12.32 ± 0.06	3.83 ± 0.66	0.36 ± 0.00	70.57 ± 0.16	4.35 ± 0.03	27.96 ± 0.10
12	38.63 ± 1.25	12.68 ± 0.72	0.36 ± 0.00	9.88 ± 0.15	73.94 ± 0.42	12.32 ± 0.72	3.32 ± 0.12	0.35 ± 0.00	70.03 ± 0.80	4.29 ± 0.09	27.56 ± 0.04
13	39.42 ± 1.85	12.72 ± 0.11	0.37 ± 0.00	9.79 ± 0.14	74.05 ± 0.63	12.34 ± 0.11	3.49 ± 0.01	0.34 ± 0.00	69.99 ± 0.24	4.40 ± 0.05	28.06 ± 0.18

**Table 3:** Coded second-order regression coefficients, determination coefficients (R^2^ and R^2^_adj_), lack of fit, and *p* values of the fitted models on loading capacity (LC), encapsulation efficiency (EE), encapsulation yield (EY), and tannin content (TC) dependent variables.

		LC	EE	EY	TC
Constant	β_0_	12.292 ***	73.484 ***	38.749 ***	98.665 ***
Linear	β_1_	5.193 ***	0.773 *	1.129 *	43.613 ***
	β_2_	−0.188 **	−1.098 **	5.366 ***	−1.863 ***
Quadratic	β_11_	−0.688 ***	−1.947 ***	−1.181 *	−2.552 ***
	β_22_	0.197 **	0.986 *	0.090	1.101 **
Interaction	β_12_	−0.068	0.124	0.774	−0.050
R^2^		0.995	0.790	0.778	0.997
R_Ad_^j2^		0.994	0.728	0.745	0.997
Lack of Fit		0.224	0.103	0.06	0.088
*p* value		<0.0001	<0.0001	<0.0001	<0.0001

*, **, *** significantly different at *p* > 0.05, *p* < 0.01, and *p* < 0.001, respectively. β0: constant; β1: coefficient of the linear effect of core:shell; β2: coefficient of the linear effect of MD:GA; β11: coefficient of the quadratic effect of core:shell; β22: coefficient of the quadratic effect of MD:GA; β12: interaction coefficient of core:shell and MD:GA.

**Table 4:** Kinetic release parameters of not encapsulated and encapsulated extract in various elution media.

Sample	Model Name	Rumen		Abomasum		Intestine	
Not encapsulated QP	Zero order	y = 1.5412x + 26.217	R^2^ = 0.4651	y = 1.0158x + 31.752	R^2 =^ 0.7998	y = 1.382x + 28.792	R^2^ = 0.5487
	First order	y = 0.0109x + 1.8586	R^2^ = 0.5809	y = 0.0104x + 1.8798	R^2^ = 0.6501	y = 0.0109x + 1.8586	R^2^ = 0.582
	Higuchi	y = 10.375x + 15.239	R^2^ = 0.7464	y = 9.9651x + 12.897	R^2^ = 0.8086	y = 11.923x + 8.8506	R^2^ = 0.8054
	Kors-Peppas	y = 0.7485x + 0.9566	R^2^ = 0.3668	y = 0.7397x + 0.9284	R^2^ = 0.3766	y = 0.7485x + 0.9569	R^2^ = 0.3665
Encapsulated QP	Zero order	y = 0.9864x + 8.7403	R^2^ = 0.5947	y = 1.4774x + 15.742	R^2^ = 0.5948	y = 1.5528x + 27.204	R^2^ = 0.4845
	First order	y = 0.0052x + 1.9588	R^2^ = 0.6174	y = −0.009x + 1.9218	R^2^ = 0.6430	y = 0.0114x + 1.8545	R^2^ = 0.6327
	Higuchi	y = 6.1999x + 2.6061	R^2^ = 0.8318	y = 9.3799x + 6.364	R^2^ = 0.8490	y = 10.242x + 6.573	R^2^ = 0.7463
	Kors-Peppas	y = 0.8239x + 0.5534	R^2^ = 0.6539	y = 0.8083x + 0.7743	R^2^ = 0.5047	y = 0.7111x + 0.992	R^2^ = 0.3283

## Supplementary Materials

The following are available online at https://www.mdpi.com/article/10.3390/foods10040881/s1, Table S1: Phenolic profile by UHPLC-QTOF of the QP.

## References

[1] Carreño D., Hervás G., Toral P.G., Belenguer A., Frutos P. (2015). Ability of different types and doses of tannin extracts to modulate in vitro ruminal biohydrogenation in sheep. Anim. Feed Sci. Technol..

[2] Vasta V., Daghio M., Cappucci A., Buccioni A., Serra A., Viti C., Mele M. (2019). Invited review: Plant polyphenols and rumen microbiota responsible for fat. acid biohydrogenation, fiber digestion, and methane emission: Experimental evidence and methodological approaches. J. Dairy Sci..

[3] Jerónimo E., Pinheiro C., Lamy E., Dentinho M.T., Sales-Baptista E., Lopes O., Silva F., Combs C.A. (2016). Tannins in ruminant nutrition: Impact on animal performance and quality of edible products. Tannins: Biochemistry, Food Sources and Nutritional Properties.

[4] Vasta V., Makkar H.P., Mele M., Priolo A. (2008). Ruminal biohydrogenation as affected by tannins in vitro. Br. J. Nutr..

[5] Ianni A., Innosa D., Martino C., Bennato F., Martino G. (2019). Compositional characteristics and aromatic profile of caciotta cheese obtained from Friesian cows fed with a dietary supplementation of dried grape pomace. J. Dairy Sci..

[6] Adejoro F.A., Hassen A., Thantsha M.S. (2019). Characterization of starch and gum arabic-maltodextrin microparticles encapsulating acacia tannin extract and evaluation of their potential use in ruminant nutrition. Asian-Australas. J. Anim. Sci..

[7] Adejoro F.A., Hassen A., Thantsha M.S. (2018). Preparation of acacia tannin loaded lipid microparticles by solid-in-oil-in-water and melt dispersion methods, their characterization and evaluation of their effect on ruminal gas production in vitro. PLoS ONE.

[8] Tolve R., Galgano F., Caruso M.C., Tchuenbou-Magaia F.L., Condelli N., Favati F., Zhang Z. (2016). Encapsulation of health-promoting ingredients: Applications in foodstuffs. Int. J. Food Sci. Nutr..

[9] Tolve R., Condelli N., Caruso M.C., Genovese F., di Renzo G.C., Mauriello G., Galgano F. (2019). Preparation and characterization of microencapsulated phytosterols for the formulation of functional foods: Scale up from laboratory to semi-technical production. Food Res. Int..

[10] Aliakbarian B., Sampaio F.C., de Faria J.T., Pitangui C.G., Lovaglio F., Casazza A.A., Converti A., Perego P. (2018). Optimization of spray drying microencapsulation of olive pomace polyphenols using response surface methodology and artificial neural network. LWT Food Sci. Technol..

[11] Ćujić-Nikolić N., Stanisavljević N., Šavikin K., Kalušević A., Nedović V., Samardžić J., Janković T. (2019). Chokeberry polyphenols preservation using spray drying: Effect of encapsulation using maltodextrin and skimmed milk on their recovery following in vitro digestion. J. Microencapsul..

[12] Papoutsis K., Golding J.B., Vuong Q., Pristijono P., Stathopoulos C.E., Scarlett C.J., Bowyer M. (2018). Encapsulation of citrus by-product extracts by spray-drying and freeze-drying using combinations of maltodextrin with soybean protein and ι-Carrageenan. Foods.

[13] Mehran M., Masoum S., Memarzadeh M. (2020). Microencapsulation of Mentha spicata essential oil by spray drying: Optimization, characterization, release kinetics of essential oil from microcapsules in food models. Ind. Crops Prod..

[14] Singleton V.L., Rossi J.A. (1965). Colorimetry of total phenolics with phosphomolybdic-phosphotungstic acid reagents. Am. J. Enol. Vitic..

[15] Dewanto V., Wu X., Adom K.K., Liu R.H. (2002). Thermal processing enhances the nutritional value of tomatoes by increasing total antioxidant activity. J. Agric. Food Chem..

[16] Caruso M.C., Galgano F., Grippo A., Condelli N., di Cairano M., Tolve R. (2019). Assay of healthful properties of wild blackberry and elderberry fruits grown in Mediterranean area. J. Food Meas. Charact..

[17] Rocchetti G., Lucini L., Chiodelli G., Giuberti G., Gallo A., Masoero F., Trevisan M. (2017). Phenolic profile and fermentation patterns of different commercial gluten-free pasta during in vitro large intestine fermentation. Food Res. Int..

[18] Rocchetti G., Lucini L., Rodriguez J.M.L., Barba F.J., Giuberti G. (2019). Gluten-free flours from cereals, pseudocereals and legumes: Phenolic fingerprints and in vitro antioxidant properties. Food Chem..

[19] Zanoni F., Primiterra M., Angeli N., Zoccatelli G. (2020). Microencapsulation by spray-drying of polyphenols extracted from red chicory and red cabbage: Effects on stability and color properties. Food Chem..

[20] Xu Y., Hanna M.A. (2006). Electrospray encapsulation of water-soluble protein with polylactide: Effects of formulations on morphology, encapsulation efficiency and release profile of particles. Int. J. Pharm..

[21] Association of Official Agricultural Chemists (AOAC) (2000). Official Methods of Analysis of the Association of Official Analytical Chemists International.

[22] Navarro-Flores M.J., Ventura-Canseco L.M.C., Meza-Gordillo R., Ayora-Talavera T.d.R., Abud-Archila M. (2020). Spray drying encapsulation of a native plant extract rich in phenolic compounds with combinations of maltodextrin and non-conventional wall materials. J. Food Sci. Technol..

[23] Jafari S., Soleimani M., Badinezhad M. (2021). Application of different mathematical models for further investigation of in vitro drug release mechanisms based on magnetic nano-composite. Polym. Bull..

[24] Marsal A., Cuadros S., Manich A.M., Izquierdo F., Font J. (2017). Reduction of the formaldehyde content in leathers treated with formaldehyde resins by means of plant polyphenols. J. Clean. Prod..

[25] Tolun A., Altintas Z., Artik N. (2016). Microencapsulation of grape polyphenols using maltodextrin and gum arabic as two alternative coating materials: Development and characterization. J. Biotechnol..

[26] Tonon R.V., Brabet C., Pallet D., Brat P., Hubinger M.D. (2009). Physicochemical and morphological characterisation of açai (Euterpe oleraceae Mart.) powder produced with different carrier agents. Int. J. Food Sci. Technol..

[27] Cilek B., Luca A., Hasirci V., Sahin S., Sumnu G. (2012). Microencapsulation of phenolic compounds extracted from sour cherry pomace: Effect of formulation, ultrasonication time and core to coating ratio. Eur. Food Res. Technol..

[28] Premi M., Sharma H.K. (2017). Effect of different combinations of maltodextrin, gum arabic and whey protein concentrate on the encapsulation behavior and oxidative stability of spray dried drumstick (Moringa oleifera) oil. Int. J. Biol. Macromol..

[29] Carmona P.A.O., Garcia L.C., de Aquino Ribeiro J.A., Valadares L.F., de Figueiredo Marçal A., de França L.F., Mendonça S. (2018). Effect of solids content and spray-drying operating conditions on the carotenoids microencapsulation from pressed palm fiber oil extracted with supercritical CO_2_. Food Bioprocess. Technol..

[30] Barbosa-Cánovas G.V., Juliano P. (2005). Compression and compaction characteristics of selected food powders. Adv. Food Nutr. Res..

[31] Mahdi A.A., Mohammed J.K., Al-Ansi W., Ghaleb A.D., Al-Maqtari Q.A., Ma M., Ahmed I.S., Wang H. (2020). Microencapsulation of fingered citron extract with gum arabic, modified starch, whey protein, and maltodextrin using spray drying. Int. J. Biol. Macromol..

[32] Kang Y.R., Lee Y.K., Kim Y.J., Chang Y.H. (2019). Characterization and storage stability of chlorophylls microencapsulated in different combination of gum Arabic and maltodextrin. Food Chem..

[33] Mohd Nawi N., Muhamad I.I., Mohd Marsin A. (2015). The physicochemical properties of microwave-assisted encapsulated anthocyanins from Ipomoea batatas as affected by different wall materials. Food Sci. Nutr..

[34] Rodríguez-Hernández G.R., González-García R., Grajales-Lagunes A., Ruiz-Cabrera M.A., Abud-Archila M. (2005). Spray-drying of cactus pear juice (Opuntia streptacantha): Effect on the physicochemical properties of powder and reconstituted product. Dry. Technol..

[35] Nale Z., Tontul I., Aşçi Arslan A., Sahin Nadeem H., Kucukcetin A. (2018). Microbial viability, physicochemical and sensory properties of kefir microcapsules prepared using maltodextrin/Arabic gum mixes. Int. J. Dairy Technol..

[36] Corrêa-Filho L.C., Lourenço M.M., Moldão-Martins M., Alves V.D. (2019). Microencapsulation of β-Carotene by spray drying: Effect of wall material concentration and drying inlet temperature. Int. J. Food Sci..

[37] Derringer G., Suich R. (1980). Simultaneous optimization of several response variables. J. Qual. Technol..

[38] Augustin M.A., Sanguansri L., Margetts C., Young B.J.F.A. (2001). Microencapsulating food ingredients. Food Aust..

[39] Tan C., Xie J., Zhang X., Cai J., Xia S. (2016). Polysaccharide-based nanoparticles by chitosan and gum arabic polyelectrolyte complexation as carriers for curcumin. Food Hydrocoll..

[40] Norkaew O., Thitisut P., Mahatheeranont S., Pawin B., Sookwong P., Yodpitak S., Lungkaphin A. (2019). Effect of wall materials on some physicochemical properties and release characteristics of encapsulated black rice anthocyanin microcapsules. Food Chem..

[41] Kar S., Kundu B., Reis R.L., Sarkar R., Nandy P., Basu R., Das S. (2019). Curcumin ameliorates the targeted delivery of methotrexate intercalated montmorillonite clay to cancer cells. Eur. J. Pharm. Sci..

[42] Martínez J.P.Q., Ruiz J.C.R., Campos M.R.S. (2018). Release kinetic studies of *Stevia rebaudiana* extract capsules from sodium alginate and inulin by ionotropic gelation. Adv. Mater. Sci. Eng..

